# Antioxidant and antibacterial activity of *Apis laboriosa* honey against *Salmonella enterica* serovar Typhimurium

**DOI:** 10.3389/fnut.2023.1181492

**Published:** 2023-05-11

**Authors:** Weihua Tan, Yuanyuan Tian, Qingya Zhang, Siwei Miao, Wenrong Wu, Xiaoqing Miao, Haiou Kuang, Wenchao Yang

**Affiliations:** ^1^College of Animal Science (College of Bee Science), Fujian Agriculture and Forestry University, Fuzhou, Fujian, China; ^2^Bee Product Processing and Application Research Center of the Ministry of Education, Fuzhou, Fujian, China; ^3^College of Food Science, Fujian Agriculture and Forestry University, Fuzhou, Fujian, China; ^4^M.X.’s Expert Workstation, Pu’er, Yunnan, China; ^5^Research Institute of Eastern Honeybee, Yunnan Agricultural University, Kunming, Yunnan, China

**Keywords:** *Apis laboriosa* honey, antioxidant activity, antibacterial activity, *Salmonella* Typhimurium, proteomics

## Abstract

*Salmonella enterica* serovar Typhimurium (*S.* Typhimurium) is a common food-borne pathogen that commonly causes gastroenteritis in humans and animals. *Apis laboriosa* honey (ALH) harvested in China has significant antibacterial activity against *Staphylococcus aureus*, *Escherichia coli,* and *Bacillus subtilis*. We hypothesize that ALH has antibacterial activity against *S.* Typhimurium. The physicochemical parameters, minimum inhibitory and bactericidal concentrations (MIC and MBC) and the possible mechanism were determined. The results showed that there were significantly different physicochemical parameters, including 73 phenolic compounds, among ALH samples harvested at different times and from different regions. Their antioxidant activity was affected by their components, especially total phenol and flavonoid contents (TPC, TFC), which had a high correlation with antioxidant activities except for the O_2_- assay. The MIC and MBC of ALH against *S.* Typhimurium were 20–30% and 25–40%, respectively, which were close to those of UMF5+ manuka honey. The proteomic experiment revealed the possible antibacterial mechanism of ALH1 at IC_50_ (2.97%, w/v), whose antioxidant activity reduced the bacterial reduction reaction and energy supply, mainly by inhibiting the citrate cycle (TCA cycle), amino acid metabolism pathways and enhancing the glycolysis pathway. The results provide a theoretical basis for the development of bacteriostatic agents and application of ALH.

## 1. Introduction

Honey is a naturally sweet substance made from the nectar of blossoms or the excretions of plant-sucking insects living on parts of plants by honey bees. Honey is composed of carbohydrates (90–95% of dry mass), approximately 17% water, and minor components (approximately 3% in total), for instance, proteins, amino acids, organic acids, vitamins, minerals, polyphenols, and volatile compounds, which content vary depending on the floral source, type of bee, environmental conditions, and/or extraction, processing, and storage conditions of packaged honey (1). Honey is used not only as a natural food but also as a treatment for diseases and wounds (1–3). Furthermore, honey has antioxidant (4), antibacterial (5), anticancer (6), anti-inflammatory (7), antivirus (2), and prebiotic activities (1).

The antioxidant activity of honey is a popular topic. Different antioxidant activities of honey samples are variable depending on their botanical sources (8), geographic regions (9), processing and storage (10) and determination assays (11), which include diphenyl-1-picrylhydrazyl (DPPH), ferric reducing antioxidant power (FRAP), oxygen radical absorbance capacity (ORAC), 2,2-azinobis (3-ethylbenzothiazoline-6-sulfonic acid) diammonium salt (ABTS), 6-hydroxy-2,5,7,8-tetramethylchroman-2-carboxylic acid (Trolox)-equivalent antioxidant capacity (TEAC), ascorbic acid content and different enzyme assays, such as catalase (CAT), glutathione peroxidase (GPO), and superoxide dismutase (SOD) assays. Phenolic compounds are mainly responsible for the antioxidant activity of honey (8, 12–15). Total flavonoid content, total water-soluble vitamins, mineral content and proteins also contribute to the antioxidant activity of honey (8, 9, 12). There are only a few reports about the antioxidant activities of *Apis laboriosa* honey (ALH), a wild honey made from the nectar of blossoms or the excretions of plant-sucking insects living on parts of plants by *Apis laboriosa* workers, which also vary by harvesting season, region and botanical source (16).

On the other hand, the antibacterial activity of honey has been widely studied. Honey has potential antibacterial effects against a wide range of bacteria and even against several antibiotic-resistant bacteria (17). The osmotic pressure, hydrogen peroxide content, phytochemical factors, low pH, phenolic acid level and flavonoid compounds of honey contribute to its antibacterial effect (18, 19). Phenolic compounds in honey play an important role in its biological and antibacterial abilities (20). At the same time, honey may contain some unknown components that inhibit the growth of bacteria (21–23). Our previous research demonstrated that ALH harvested in China has significant antibacterial activity against *Staphylococcus aureus, Escherichia coli,* and *Bacillus subtilis* when diluted to 90% moisture content (24). It was reported that ALH from Nepal has an antibacterial effect against *S. aureus* and *E. coli* (25). The differences in the antibacterial activity of ALH were attributed to differences in its botanical sources and entomological proteins (24). The antibacterial activity of ALH against *Salmonella enterica* serovar Typhimurium (*S.* Typhimurium) is unknown.

*Salmonella* Typhimurium is a common food-borne pathogen that commonly causes gastroenteritis in humans and animals. The replication of *S.* Typhimurium in the intestinal tract is sustained by the secretion of effector proteins by type III secretion systems to trigger an inflammatory response without the engagement of innate immune receptors (26). *S.* Typhimurium was listed as “the most threatening to public health” by the Centers for Disease Control and Prevention due to its frequent adulteration of beef and poultry food products and its association with multidrug resistance (27, 28). Therefore, developing new strategies to inhibit this bacterium is urgent. Previous reports showed that silver (Ag) and citric acid coated iron oxide (Fe_3_O_4_) nanoparticles (29), olive oil polyphenol extract (30), cocktails of phages targeting multiple host receptors (31), novel deoxy-tetradeuterio-curcumin derivative (32), linalool nanoemulsions (33), zinc oxide nanoparticles (34), etc., had antibacterial activities against *S.* Typhimurium. At present, research data on the antibacterial ability and antibacterial mechanisms of ALH against *S.* Typhimurium are limited.

In this study, the antioxidant and antibacterial activities and possible antibacterial mechanism of ALH was investigated, which will provide an alternative bacteriostatic strategy against *S.* Typhimurium.

## 2. Materials

### 2.1. Honey samples, bacterial strain, and chemicals

The study was performed between March 2021 and October 2022 at the Bee Product Processing and Application Research Center of the Ministry of Education (Fuzhou) at Fujian Agricultural and Forestry University.

ALH samples were harvested from Yunnan Province, China. The harvesting location and date information are listed in Table 1. Manuka honey (MH: UMF5+, 10+, and 15+: MH1, MH2, and MH3, respectively), which UMF data means the antibacterial activity of honey is the same as that of phenol 5, 10, and 15% aqueous solution respectively, was purchased from Comvita ® News Land in August 2019 and mailed to our lab. These honey samples were stored in a refrigerator at 4°C until testing.

**Table 1:** Information of the ALH samples.

Samples	Location (in Yunnan Province, China)	Date
ALH1	Bingzhongluo, Gongshan County, Nujiang Prefecture	April 2019
ALH2	Menglian County, Pu’er City	April 2018
ALH3	Liming Village, Fengping Town, Dehongmang City	May 2021
ALH4	Lu Zhang Zhen Luo Ma Cun, Hushui City, Nujiang Prefecture	June 2021
ALH5	Sudian, Yingjiang County, Mang City, Dehong Prefecture	March 2020

The bacterial strain *Salmonella enterica* serovar Typhimurium (ATCC14028) (*S.* Typhimurium) used in this experiment was purchased from Guangdong Huankai Microbial Sci. & Tech. Co., Ltd., Guangzhou, China.

Chemicals of 1,1-diphenyl-2-trinitrophenylhydrazine, 6-hydroxy-2,5,7,8-tetramethyltryptophan-2-carboxylic acid, 2,2-diazodi (3-ethyl-benzothiazole-6-sulfonic acid) diammonium salt, phenazine methyl sulfate β-Nicotinamide adenine dinucleotide disodium salt, nitrotetrazolium chloride blue, 2,4,6-tripyridyl triazine, and analytical methanol were purchased from Shanghai McLean Biochemical Technology Co., Ltd. Shanghai, China. Chemicals of ferric chloride, hydrochloric acid, acetic acid, sodium acetate, sodium dihydrogen phosphate and disodium hydrogen phosphate were purchased from Sinopharm Chemical Reagent Co., Ltd. Shanghai, China. LB broth medium was purchased from Bioengineering (Shanghai) Co., Ltd. Shanghai, China. TTC nutritional agar, folinol and rutin were purchased from Beijing Solebar Technology Co., Ltd. Beijing, China. Gallic acid was purchased from Shanghai Yuanye Co., Ltd. Shanghai, China. Hieff UNICON Universal Blue qPCR SYBR Green Master Mix was purchased from Yeasen Biotechnology Co., Ltd. Shanghai, China.

### 2.2. Physicochemical properties of ALH samples

Determinations of the moisture, ash, pH, glucose, fructose, sucrose and 5-HMF in ALH were performed according to the method of our earlier report (24). The total phenol and flavonoid contents were determined according to a previous method (35). The data were calculated by the dry weight of ALH.

The phenolic components were determined by untargeted metabolomics at Novogene Co., Ltd. (Beijing, China). The honey samples (100 μl) were placed in EP tubes and resuspended in prechilled 80% methanol by vortexing well. Then, the samples were incubated on ice for 5 min and centrifuged at 15,000 *g* and 4°C for 20 min. The supernatant was diluted to a final concentration containing 53% methanol by LC–MS grade water and then centrifuged at 15,000 *g* and 4°C for 20 min. The supernatant was injected into the LC–MS/MS system for analysis. UHPLC–MS/MS analyses were performed using a Vanquish UHPLC system (Thermo Fisher, Germany) coupled with an Orbitrap Q Exactive TM HF-X mass spectrometer (Thermo Fisher, Germany). Samples were injected onto a Hypersil Gold column (100 mm × 2.1 mm, 1.9 μm) using a 17-min linear gradient at a flow rate of 0.2 ml/min. The eluents for the positive polarity mode were eluent A (0.1% formic acid in water) and eluent B (methanol). The eluents for the negative polarity mode were eluent A (5 m ammonium acetate, pH 9.0) and eluent B (methanol). The solvent gradient was set as follows: 2% B for 1.5 min, 2–85% B for 3 min, 85–100% B for 10 min, 100–2% B for 10.1 min, and 2% B for 12 min. A Q Exactive TM HF-X mass spectrometer was operated in positive/negative polarity mode with a spray voltage of 3.5 kV, capillary temperature of 320°C, sheath gas flow rate of 35 psi, aux gas flow rate of 10 l/min, S-lens RF level of 60, and aux gas heater temperature of 350°C. The raw data files were obtained for further analysis.

### 2.3. Antioxidant activity of ALH

The DPPH radical scavenging activity and FRAP total antioxidant capacity were determined according to a previous method (36) at 517 and 593 nm, respectively. Trolox was used as the standard, and the result is expressed in Trolox equivalents, mg TE/100 g.

According to a previous method (37), ABTS cation radical scavenging activity was determined at 734 nm. Trolox was used as the standard and expressed as the result using its equivalent, mg TE/100 g.

The reduction ability of the ALH solution in the system was determined by detecting the absorbance value with an ultraviolet spectrophotometer (T6, Beijing Puxi General Instrument Co., Ltd. Beijing, China) at 560 nm, with NADH-PMS-NBT used as the superoxide anion (O_2_·-) generation system (38).

All antioxidant activities were expressed by the dry weight of ALH.

### 2.4. Antibacterial activity of ALH against *Salmonella* Typhimurium

The bacterial suspension, which was adjusted to 1 × 10^8^ CFU/ml with physiological saline, was added to the 96-well plate (10 μl). Then, 190 μl of culture medium (ALH and broth culture) was added and mixed well. It was placed in a shaker incubator at 80 r/min at 37°C for 24 h.

#### 2.4.1. Minimum inhibitory concentration determination

Minimum inhibitory concentration (MIC) determination was performed according to a previous method (39). The ALH and broth culture medium were mixed evenly and prepared at different concentrations of 0, 5, 10, 15, 20, 25, 30, 35, and 40% (dry weight of honey/volume of mixture); other ALH concentrations for antibacterial activity determination were the same. The mixed liquid after shaking was filtered with a 0.22 μm filter membrane for further determination.

#### 2.4.2. Minimum bactericidal concentration (MBC) determination

The liquid (200 μl) in the 96-well plate was transferred to LB solid medium, incubated at 37°C for 24 h, and the growth of the colony in the solid culture was observed. MBC is the lowest honey concentration without colony growth (39).

#### 2.4.3. IC_50_ of ALH1 determination

The honey sample ALH1 has medium IC_50_ against *S.* Typhimurium (Table 2). ALH1 was selected for further antibacterial mechanism experiment.

**Table 2:** Minimum inhibitory concentration (MIC) and minimum bactericidal concentration (MBC) of honey samples against *S. typhimurium*.

Samples	*S.* typhimurium	
	MIC (%)	MBC (%)
ALH1	25	30
ALH2	30	40
ALH3	20	25
ALH4	30	35
ALH5	25	30
MH1	25	35
MH2	15	20
MH3	10	10

Broth medium with 0, 2, 4, 6, and 8% (w/v) ALH1 was prepared and filtered through a 0.22 μm filter. Their inhibition rates were calculated according to their optical densities. The IC_50_ was calculated using GraphPad Prism 8.4.3 for Windows (GraphPad Software, Inc.) according to the inhibition rates of different concentrations of ALH1 against *S.* Typhimurium.

### 2.5. Label-free proteomic of *Salmonella* Typhimurium treated with ALH1

*Salmonella* Typhimurium was cultured as the treatment group (IC_50_ of ALH1, 2.97% w/v) and the control group (no ALH1). After culturing, *S.* Typhimurium was collected and snap-frozen in liquid nitrogen and stored at −80°C for subsequent experiments. The proteins were determined using LC–MS–MS with a Q Exactive HF-X mass spectrometer (Thermo Fisher, Germany) with a Nanospray Flex™ electrospray ionization (ESI) source by Novgene Biotech Co., Ltd., China.

### 2.6. Relative gene expression of *Salmonella* Typhimurium treated with ALH1

*Salmonella* Typhimurium was cultured with IC_50_ ALH1. RNA was extracted using the TransZol UP kit, and its concentration and purity were determined by an ultramicro UV spectrophotometer (NanoDrop One; Thermo Fisher Scientific, United States). The relative expression levels of gene-coded proteins in protein interactions in the citrate cycle (TCA cycle) and bacterial chemotaxis (*icdA*, *gltA*, *fumC*, *nuoC*, *sucB*, *lpdA*, *motA*, *FliG*, *motB*, *pgi*) were determined using RT-PCR assays. cDNA synthesis was performed using the HiScript II Q RT SuperMix for qPCR (+gDNA wiper) kit. The internal reference gene was *5S* (40). Their primer sequences were designed using Primer Premier 5.0 (Primer) and were listed in Supplementary Table S1, which synthesis were performed by Sangon Biotech (Shanghai, China) Co., Ltd. These primers were identified by RT-PCR procedure was performed as previously described (41). Finally, a SYBR mixture was used for gene expression quantification.

### 2.7. Data analysis

All experiments were performed in triplicate. The experimental results were expressed as the mean ± standard error. Percentages (*p*) were then transformed to arc sin (degree) values (according to the formula arc sin√p) before ANOVA. One-way ANOVA was used to analyze the significance differences using GraphPad Prism 8.0.2 for Windows (GraphPad Software, Inc.). The correlations between TPC, TFC and antioxidant activity were analyzed using IBM SPSS Statistics 26.0 (IBM). The relative gene expression was expressed by the ratio of the target gene to the internal reference gene.

The raw data files of phenolic compounds were processed using Compound Discoverer 3.1 (CD3.1, Thermo Fisher) to perform peak alignment, peak picking, and quantitation for each metabolite. The main parameters were set as follows: retention time tolerance of 0.2 min, actual mass tolerance of 5 ppm, signal intensity tolerance of 30%, signal/noise ratio of 3, and minimum intensity. The peak intensities were normalized to the total spectral intensity. The normalized data were used to predict the molecular formula based on additive ions, molecular ion peaks and fragment ions. These peaks were matched with the mzCloud^1^, mzVault and MassList databases to obtain accurate qualitative and relative quantitative results (*r*^2^ ≥ 0.99). Statistical analyses were performed using the statistical software R (R-3.4.3), Python (Python V2.7.6) and CentOS (CentOS release 6.6). Compounds whose CVs of relative peak areas in QC samples were greater than 30% were removed, and finally, the metabolites’ identification and relative quantification results were obtained. All the phenolic components were screened according to a previous method (42).

The spectra of proteins obtained from LC–MS/MS were searched against the UniProt database blasted with the NCBI database by Proteome Discoverer 2.2 (Thermo) with a credibility of more than 99% Peptide Spectrum Matches. Protein identification, differential protein definition (fold change ≥2 or ≤0.5; *p* < 0.05), GO term enrichment, KEGG pathway enrichment and protein–protein interactions (PPI, interaction score 0.900) were explored as described in our previous report (43).

## 3. Results

### 3.1. Physicochemical properties of ALH samples

The physicochemical parameters of the ALH samples were shown in Table 3. The moisture, ash, pH, glucose, fructose, sucrose, 5-HMF contents, total phenols and total flavonoids in these samples were significantly different and are labeled with different lowercase letters. The physicochemical parameters of MH1, MH2, and MH3 were the same as those in our previous report (24). There were 73 phenolic compounds (Table 4), which spectrogram are shown in Supplementary Figure S1.

**Table 3:** Physicochemical parameters of the ALH samples.

	Moisture (%)	Ash (%)	Glucose (%)	Fructose (%)	Sucrose (%)	5-HMF (mg/kg)	pH	TPC (mg GAE/100 g)	TFC (mg RE/100 g)
ALH1	21.4 ± 0.11^c*^	0.242 ± 0.015^a^	40.98 ± 0.09^a^	39.55 ± 0.04^a^	2.66 ± 0.03^d^	1.12 ± 0.04^b^	3.89 ± 0.01^e^	55.48 ± 0.45^c^	18.99 ± 0.24^c^
ALH2	16.96 ± 0.30^d^	0.07 ± 0.029^b^	36.36 ± 0.08^b^	38.89 ± 0.08^b^	3.05 ± 0.01^c^	3.76 ± 0.04^a^	4.12 ± 0.01^c^	62.11 ± 0.45^b^	27.82 ± 0.24^b^
ALH3	23.94 ± 0.30^b^	0.077 ± 0.023^b^	32.51 ± 0.04^c^	38.07 ± 0.04^c^	3.27 ± 0.03^b^	0.31 ± 0.01^d^	4.17 ± 0.03^b^	52.54 ± 0.00^d^	12.55 ± 0.92^d^
ALH4	23.55 ± 0.11^b^	0.267 ± 0.04^a^	28.81 ± 0.13^e^	36.41 ± 0.14^d^	4.56 ± 0.03^a^	0.45 ± 0.14^cd^	4.29 ± 0.01^a^	123.16 ± 0.00^a^	51.62 ± 1.56^a^
ALH5	26.41 ± 1.02^a^	0.129 ± 0.021^b^	31.70 ± 0.13^d^	35.80 ± 0.11^e^	3.02 ± 0.02^c^	0.50 ± 0.12^c^	3.99 ± 0.00^d^	49.18 ± 0.00^e^	3.03 ± 1.28^e^

^*^Different lowercases mean significant differences in one column. All the data except moisture and pH were calculated by the dry weight of ALH.

**Table 4:** The phenolic compounds of the ALH samples.

No	Name	Formula	Molecular weight	RT (min)	m/z	Relative quantitative value					Polarity mode
						ALH1	ALH2	ALH3	ALH4	ALH5	
1	*o*-Cresol	C_7_H_8_O	108.05755	4.983	109.06485	46,411,072	205,588,392	392,346,171	250,012,462	597,793,298	Positive
2	Epinephrine	C_9_H_13_NO_3_	183.0896	5.077	184.09686	7278842.6	7040833.7	9180173.5	5717938.2	6665674.4	Positive
3	Isoproterenol	C_11_H_17_NO_3_	211.12094	5.113	212.12822	2960662.9	8562894.2	8274319.9	9023181.8	3166539.4	Positive
4	Catechol	C_6_H_6_O_2_	110.03689	5.331	109.02961	16,526,354	50,211,254	13,711,788	16,520,394	19,891,895	Negative
5	8-Hydroxyquinoline	C_9_H_7_NO	145.05282	5.346	146.06003	4.48E+09	1.152E+09	685,903,721	6.697E+09	3.054E+09	Positive
6	4-Methylphenol	C_7_H_8_O	108.05762	5.588	107.05035	7520596.3	24,629,769	12,285,330	4670787.6	53,031,596	Negative
7	2-Methoxyresorcinol	C_7_H_8_O_3_	140.04729	5.594	141.05457	155,726,929	40,860,899	32,036,326	55,223,939	30,536,637	Positive
8	4-Hexylresorcinol	C_12_H_18_O_2_	194.13077	5.685	193.1235	5124733.6	3237740.7	3316065.8	2766228.8	11,004,042	Negative
9	Vanillyl alcohol	C_8_H_10_O_3_	154.06307	5.686	153.0558	22,390,708	23,588,557	23,200,009	19,869,308	68,722,881	Negative
10	Metanephrine	C_10_H_15_NO_3_	197.10473	5.726	198.11211	7573373.5	6841019.7	7273887.5	7979091.3	11,289,806	Positive
11	2,5-Dimethylphenol	C_8_H_10_O	122.07324	6.002	121.06587	47,885,801	99,572,777	18,774,642	43,294,225	46,659,695	Negative
12	Tyrosol	C_8_H_10_O_2_	138.06813	6.004	137.06093	6088462.1	9374481.8	9984650.6	3917287.3	16,255,173	Negative
13	Eugenol	C_10_H_12_O_2_	164.08386	6.01	163.07657	25,997,185	69,434,124	2878888.8	9,868,492	4224552.2	Negative
14	l-Adrenaline	C_9_H_13_NO_3_	183.08949	6.026	184.09677	508543.59	1560795.1	213058.57	858430.7	184968.11	Positive
15	Gamma-tocopherol	C_28_H_48_O_2_	416.36511	11.816	417.37231	128,992,252	866,595,321	104,933,536	157,357,203	41,770,245	Positive
16	Pyrogallol	C_6_H_6_O_3_	126.03186	0.092	125.02458	230.96422	252.15739	226.76334	198.88683	257.62465	Negative
17	2,6-Dihydroxybenzoic acid	C_7_H_6_O_4_	154.02652	1.399	155.03378	30,095,857	36,691,962	35,431,835	18,756,819	22,451,370	Positive
18	4-Hydroxybenzoic acid	C_7_H_6_O_3_	138.03169	1.676	139.03899	724,759,011	399,345,820	295,041,509	242,252,164	314,300,303	Positive
19	2-Hydroxycinnamic acid	C_9_H_8_O_3_	164.04747	2.608	165.0547	943,405,178	2.569E+09	4.109E+09	3.782E+09	7.944E+09	Positive
20	3-Hydroxymandelic acid	C_8_H_8_O_4_	168.04233	4.918	167.035	29,569,239	42,703,871	24,186,518	32,737,877	74,965,430	Negative
21	*Trans*-cinnamic acid	C_9_H_8_O_2_	148.05252	4.984	147.04524	10,722,956	297,412,497	385,771,881	306,494,551	688,970,241	Negative
22	Protocatechuic acid	C_7_H_6_O_4_	154.02672	5.064	153.01942	90,666,643	365,257,718	116,832,436	98,351,978	120,559,255	Negative
23	Sinapinic acid	C_11_H_12_O_5_	206.05799	5.073	207.06531	7410129.3	118,011,942	115,968,197	33,618,313	91,305,690	Positive
24	Esculin	C_15_H_16_O_9_	340.07966	5.099	339.07239	3431299.7	11,977,322	4,388,673	3896422.4	2596629.4	Negative
25	Syringic acid	C_9_H_10_O_5_	198.05297	5.214	197.04567	8765554.1	15,729,303	8119483.5	10,915,284	10,337,949	Negative
26	Salicylic acid	C_7_H_6_O_3_	138.03175	5.381	137.02451	254,937,546	89,880,439	75,968,170	59,277,923	158,848,199	Negative
27	Esculetin	C_9_H_6_O_4_	178.02668	5.39	179.03397	13,240,196	68,933,250	15,749,727	6873948.2	11,513,115	Positive
28	2-Hydroxyphenylacetic acid	C_8_H_8_O_3_	152.04742	5.416	151.04012	68,304,921	20,945,876	35,183,168	10,528,109	275,050,043	Negative
29	Homovanillic acid	C_9_H_10_O_4_	182.05804	5.438	181.05077	7407175.7	49,595,796	10,573,101	522,238,714	20,055,306	Negative
30	2,4-Dihydroxybenzoic acid	C_7_H_6_O_4_	154.02673	5.458	153.01944	220,486,331	224,379,085	145,524,962	2.255E+09	428,199,984	Negative
31	P-Coumaroyl agmatine	C_14_H_20_N_4_O_2_	276.15741	5.486	275.15005	53,060,957	39,415,835	299,752,213	43,519,864	294,321,434	Negative
32	Methyl cinnamate	C_10_H_10_O_2_	162.06819	5.52	163.07547	76,956,417	66,488,588	56,355,473	41,483,266	55,603,321	Positive
33	4-Methoxycinnamic acid	C_10_H_10_O_3_	178.06298	5.583	177.05544	57,216,277	10,593,196	5510109.5	3648710.3	96,059,285	Negative
34	Methyl EudesMate	C_11_H_14_O_5_	226.08395	5.609	227.09143	23,229,666	25,330,636	12,357,191	7657217.9	21,865,592	Positive
35	Gentisic acid	C_7_H_6_O_4_	154.02661	5.643	155.03386	8894173.1	33,742,171	4471361.7	4008137.4	3435609.6	Positive
36	Aflatoxin G1	C_17_H_12_O_7_	328.05818	5.644	329.06546	5022807.9	6525337.6	22,961,236	7278374.8	5337109.7	Positive
37	Butylparaben	C_11_H_14_O_3_	194.09426	5.696	193.08699	41,152,182	7959086.1	13,145,489	27,220,128	73,923,399	Negative
38	Ferulic acid	C_10_H_10_O_4_	194.058	5.721	193.05069	37,837,845	21,141,903	10,377,211	15,910,044	27,149,521	Negative
39	Ethyl paraben	C_9_H_10_O_3_	166.06297	5.779	167.07031	21,199,760	123,205,173	45,163,463	25,201,560	23,314,289	Positive
40	*N*-*P*-coumaroyl spermidine	C_16_H_25_N_3_O_2_	291.19458	5.814	292.20181	134,242,897	82,016,708	70,794,633	99,294,110	27,244,753	Positive
41	3-Hydroxybenzoic acid	C_7_H_6_O_3_	138.03174	6.081	137.02446	290,543,911	539,383,844	212,596,390	418,695,002	184,654,920	Negative
42	Cannabidiolic acid	C_22_H_30_O_4_	358.21475	7.855	357.20746	74,305,724	35,246,765	10,370,246	1.539E+09	22,342,469	Negative
43	Anacardic acid	C_22_H_36_O_3_	326.28224	10.43	327.28925	2393541.7	7,716,783	12,710,046	3772440.8	10,882,398	Positive
44	Epigallocatechin	C_15_H_14_O_7_	612.15195	2.425	307.08325	50,032,967	109,554,639	162,790,082	560,143,740	37,420,376	Positive
45	Astilbin	C_21_H_22_O_11_	450.11677	5.322	449.1095	17,217,175	1706928.6	1101880.3	1375971.8	2264684.2	Negative
46	Rutin	C_27_H_30_O_16_	610.15447	5.429	609.14722	779,118,530	550,332,217	367,283,278	1.264E+09	298,848,161	Negative
47	Syringetin-3-*O*-glucoside	C_23_H_24_O_13_	508.12194	5.463	509.12924	38,757,335	756544.23	577247.84	16,498,042	2335932.2	Positive
48	Taxifolin	C_15_H_12_O_7_	304.05842	5.506	305.06696	2.972E+09	228,467,894	167,876,772	1.255E+09	428,898,762	Positive
49	Trifolin	C_21_H_20_O_11_	448.10039	5.509	449.10767	131,983,149	9,774,093	9338946.2	47,190,585	22,455,934	Positive
50	Quercetin-3β-d-glucoside	C_21_H_20_O_12_	464.0966	5.564	463.08929	152,586,291	59,261,195	23,830,695	58,982,334	66,558,301	Negative
51	Neohesperidin	C_28_H_34_O_15_	610.19005	5.589	609.18256	1089968.4	74,735,897	1361554.3	18,689,313	111,682,335	Negative
52	Hesperidin	C_28_H_34_O_15_	610.18954	5.602	611.1972	2645905.6	13,970,129	1271326.1	3766939.3	14,741,638	Positive
53	Myricetin	C_15_H_10_O_8_	318.03812	5.704	317.03061	5221572.2	82,555,019	62,710,883	28,867,403	31,108,913	Negative
54	Xanthohumol	C_21_H_22_O	354.15278	5.716	353.14551	5728441.5	7603788.5	5852378.8	5693288.7	6779381.7	Negative
55	Quercetin-3-*O*-beta-glucopyranosyl-6′-acetate	C_23_H_22_O_13_	506.10649	5.742	505.09912	4273830.7	4722705.5	9378763.2	171,951,165	25,540,419	Negative
56	Eriodictyol	C_15_H_12_O_6_	288.06334	5.858	287.05612	2717643.7	11,345,086	2921547.2	8291291.6	7086106.3	Negative
57	Quercetin	C_15_H_10_O_7_	302.04266	5.947	303.0499	657,762,749	779,066,791	156,514,223	2.043E+09	650,789,316	Positive
58	Luteolin	C_15_H_10_O_6_	286.04794	6.027	285.04065	25,938,408	77,343,293	21,899,826	24,748,780	257,903,239	Negative
59	Naringenin	C_15_H_12_O_5_	272.06866	6.082	271.06143	91,501,688	211,765,398	90,530,543	155,336,430	116,680,218	Negative
60	Nobiletin	C_21_H_22_O_8_	402.13738	6.086	403.14465	3,955,216	2671144.9	3315000.5	1862373.7	1677316.9	Positive
61	5,7-Dihydroxy-2-(3-hydroxy-4-methoxyphenyl)chroman-4-one	C_16_H_14_O_6_	302.07918	6.095	301.07181	5630990.2	298,831,531	11,900,160	92,765,439	305,211,731	Negative
62	Hesperetin	C_16_H_14_O_6_	302.07907	6.102	303.08636	1800398.7	116,519,169	4941709.5	36,586,122	76,778,048	Positive
63	Genistein	C_15_H_10_O_5_	270.05302	6.103	269.04575	3546717.1	962199.99	2,589,958	34,463,542	892936.56	Negative
64	Kaempferol	C_15_H_10_O_6_	286.04794	6.185	285.04062	6.397E+09	2.02E+09	514,283,859	5.043E+09	2.648E+09	Negative
65	Isorhamnetin	C_16_H_12_O_7_	316.05852	6.194	315.05118	1.964E+10	1.735E+10	1.328E+10	2.309E+10	1.738E+10	Negative
66	Diosmetin	C_16_H_12_O_6_	300.06342	6.245	301.07071	18,366,357	399,524,979	30,571,042	78,512,910	154,655,569	Positive
67	Apigenin	C_15_H_10_O_5_	270.05304	6.251	269.04575	7472132.3	656,562,405	86,542,187	23,662,195	62,117,144	Negative
68	Glycitein	C_16_H_12_O_5_	284.06842	6.855	283.06128	606717.71	18,076,781	1390592.2	742423.26	1723075.9	Negative
69	Chrysin	C_15_H_10_O_4_	254.0581	6.871	253.05087	580149.27	2250590.7	601869.39	960057.9	1186872.7	Negative
70	3′,5,7-Trihydroxy-4′-methoxyflavanone	C_16_H_14_O_6_	302.08125	6.904	303.08853	1102775.6	1224661.5	1684516.5	1286209.6	1084829.2	Positive
71	(+/−)-Equol	C_15_H_14_O_3_	242.09433	8.84	243.10161	360346.62	22,728,454	322450.82	255002.15	230697.24	Positive
72	Troxerutin	C_33_H_42_O_19_	780.18031	11.289	781.18817	4039184.9	5831039.4	10,570,822	3780738.7	6132471.8	Positive
73	Ellagic acid	C_14_H_6_O_8_	302.0066	5.616	300.9993	11492151.38	15729305.35	11563474.02	19912104.07	5657077.959	Negative

### 3.2. Antioxidant activities of ALH

The antioxidant activities of the ALH samples are shown in Table 5. The DPPḤ, ABTS·+, FRAP and superoxide anion scavenging activities of these samples were significantly different and are labeled with different lowercase letters.

**Table 5:** Antioxidant activities of the ALH samples.

	DPPḤ (mg TE/100 g)	ABTS· + (mg TE/100 g)	FRAP (mg TE/100 g)	O_2_·– (%)
ALH1	22.15 ± 0.74^d*^	36.22 ± 1.15^d^	51.60 ± 0.07^c^	83.84 ± 0.9^c^
ALH2	30.25 ± 0.51^b^	52.78 ± 1.22^b^	55.52 ± 0.05^b^	76.10 ± 0.93^d^
ALH3	25.29 ± 1.31^c^	45.51 ± 1.33^c^	47.32 ± 0.03^d^	87.21 ± 0.88^b^
ALH4	64.37 ± 0.23^a^	105.03 ± 1.14^a^	106.88 ± 0.15^a^	91.26 ± 0.34^a^
ALH5	16.69 ± 0.53^e^	32.82 ± 0.68^e^	38.54 ± 0.13^e^	81.99 ± 0.23^c^

^*^Different lowercases mean significant differences in one column. All the data were calculated by the dry weight of ALH.

The correlation ships among TPC, TFC and antioxidant activities are shown in Table 6. They were strongly correlated with DPPH free radical scavenging activity, ABTS free radical scavenging activity, and FRAP total antioxidant capacity (*p* < 0.05).

**Table 6:** Correlation ships among total phenols, total flavonoids and antioxidant activities.

	TPC	TFC	DPPH	ABTS+	FRAP	O_2_·-
TPC	1					
TFC	0.935	1				
DPPH	0.989	0.955	1			
ABTS+	0.984	0.943	0.998	1		
FRAP	0.994	0.960	0.992	0.983	1	
O_2_·-	0.621	0.421	0.604	0.596	0.619	1

### 3.3. Antibacterial activities of ALH and manuka honey against *Salmonella* Typhimurium

As shown in Table 2, the MIC and MBC of ALH against *S.* Typhimurium were 20–30% and 25–40%, respectively. They were higher than those of MH2 and MH3.

**Figure 1:**
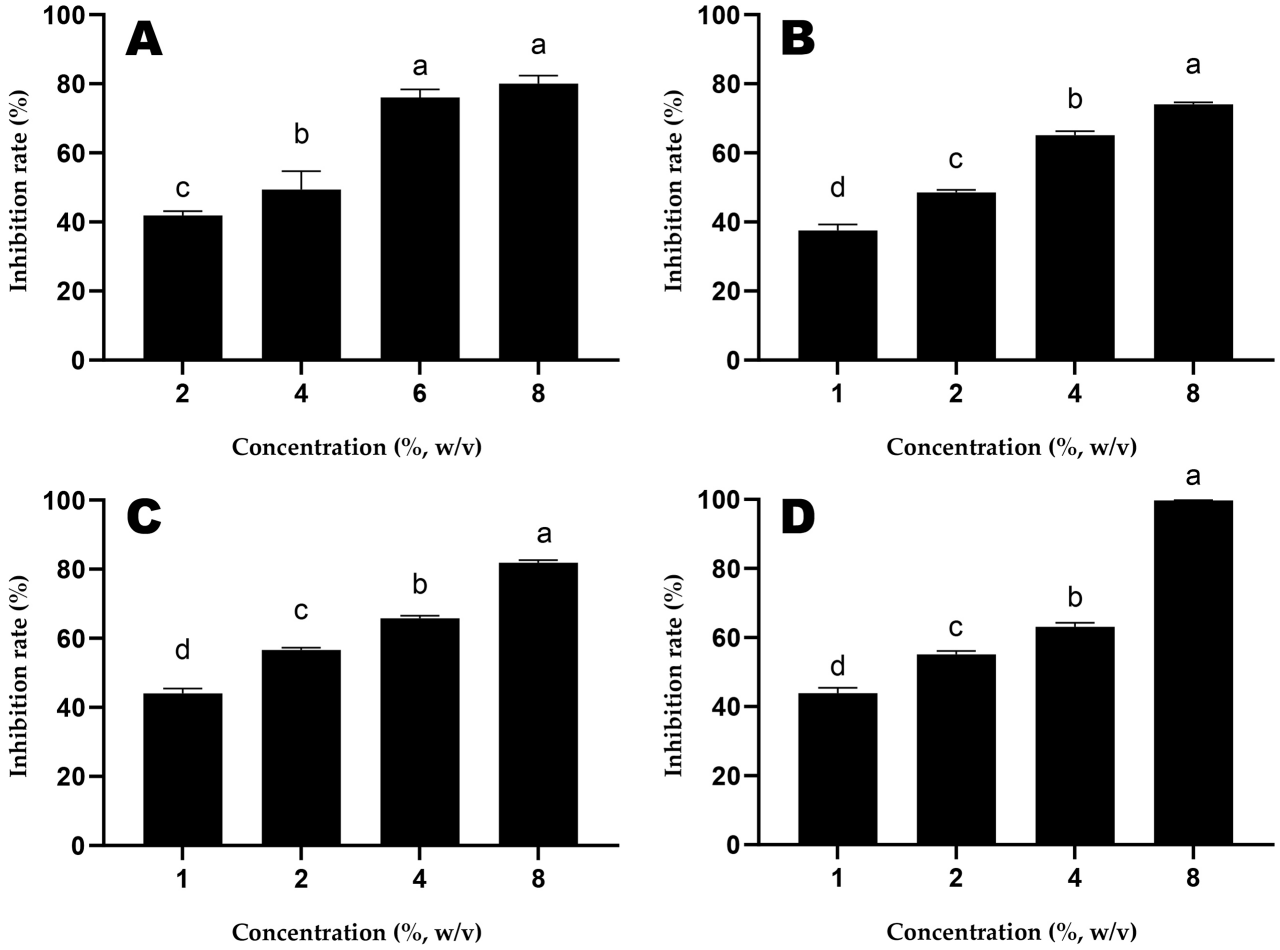
The inhibition rates of different doses of honey samples (**A**: ALH1; **B**: MH1; **C**: MH2; **D**: MH3) against *S. Typhimurium*. Different lowercases mean significant differences among inhibition rates of different doses of honey.

The inhibition rates of different doses of honey samples against *S.* Typhimurium are shown in Figure 1. The IC_50_ of ALH1 was 2.97% (w/v), 1.99% for MH1, 1.43% for MH2 and 1.50% for MH3.

### 3.4. Label-free proteomic of *Salmonella* Typhimurium treated with ALH1

**Figure 2:**
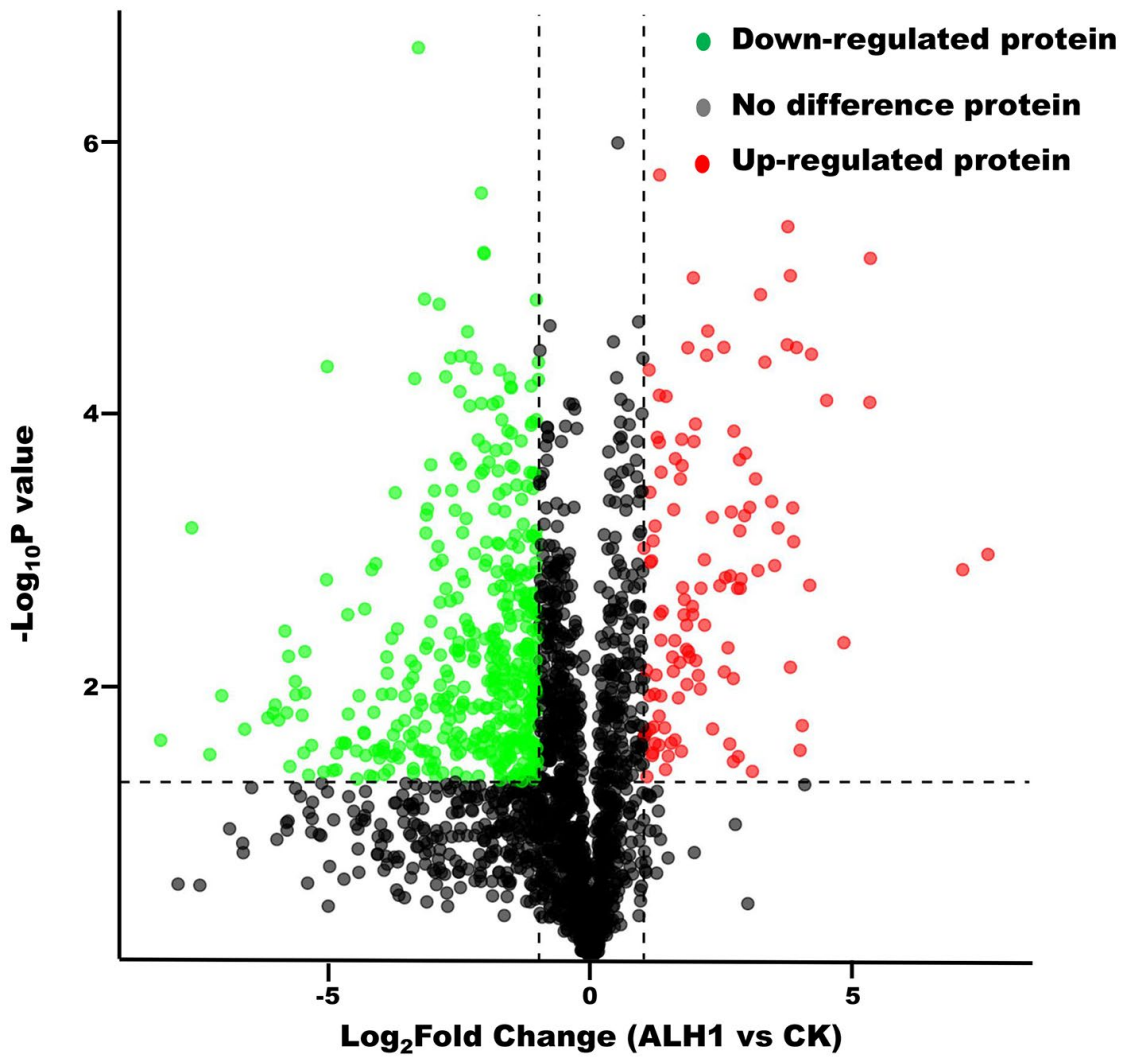
The volcano plot of proteins between *S. Typhimurium* treated with ALH1 and control groups.

There were 2,606 proteins and 708 differentially expressed proteins, which included 130 upregulated proteins (the difference in protein abundance reached 2 times or more, *p* < 0.05) and 578 downregulated proteins (the difference in protein abundance was 0.5 times or less, *p* < 0.05) (Figure 2).

**Figure 3:**
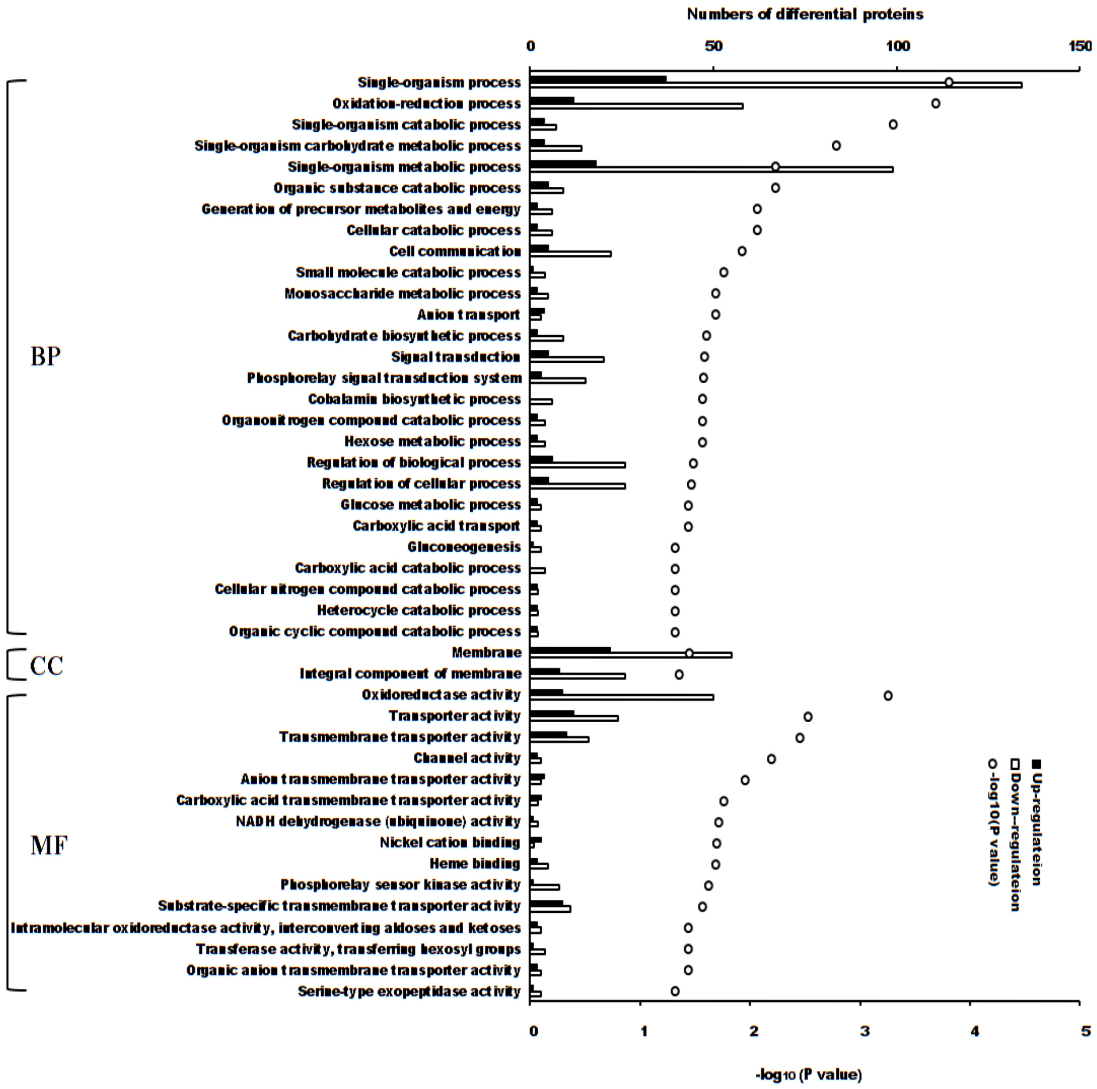
Enriched terms of Gene Ontology of differential proteins between *S. Typhimurium* treated with ALH1 and control groups. The blank and black bars mean down- or up-regulated differential proteins, repetitively. The open circle means –log_10_ (*p*-value). BP, biological process; CC, cellular component; MF, molecular function.

There were 44 enriched Gene Ontology terms of differential proteins between the treatment and control groups (*p* < 0.05, Figure 3). If the adjusted value of *p* was considered, 5 terms enriched more differential proteins (adjusted *p* < 0.05): single-organism process (171 differential proteins; *p* = 0.000149), single-organism metabolic process (117 differential proteins; *p* = 0.00574), membrane (77 differential proteins; *p* = 0.0352), oxidation–reduction process (70 differential proteins; *p* = 0.000196) and oxidoreductase activity (59 differential proteins; *p* = 0.000550).

**Figure 4:**
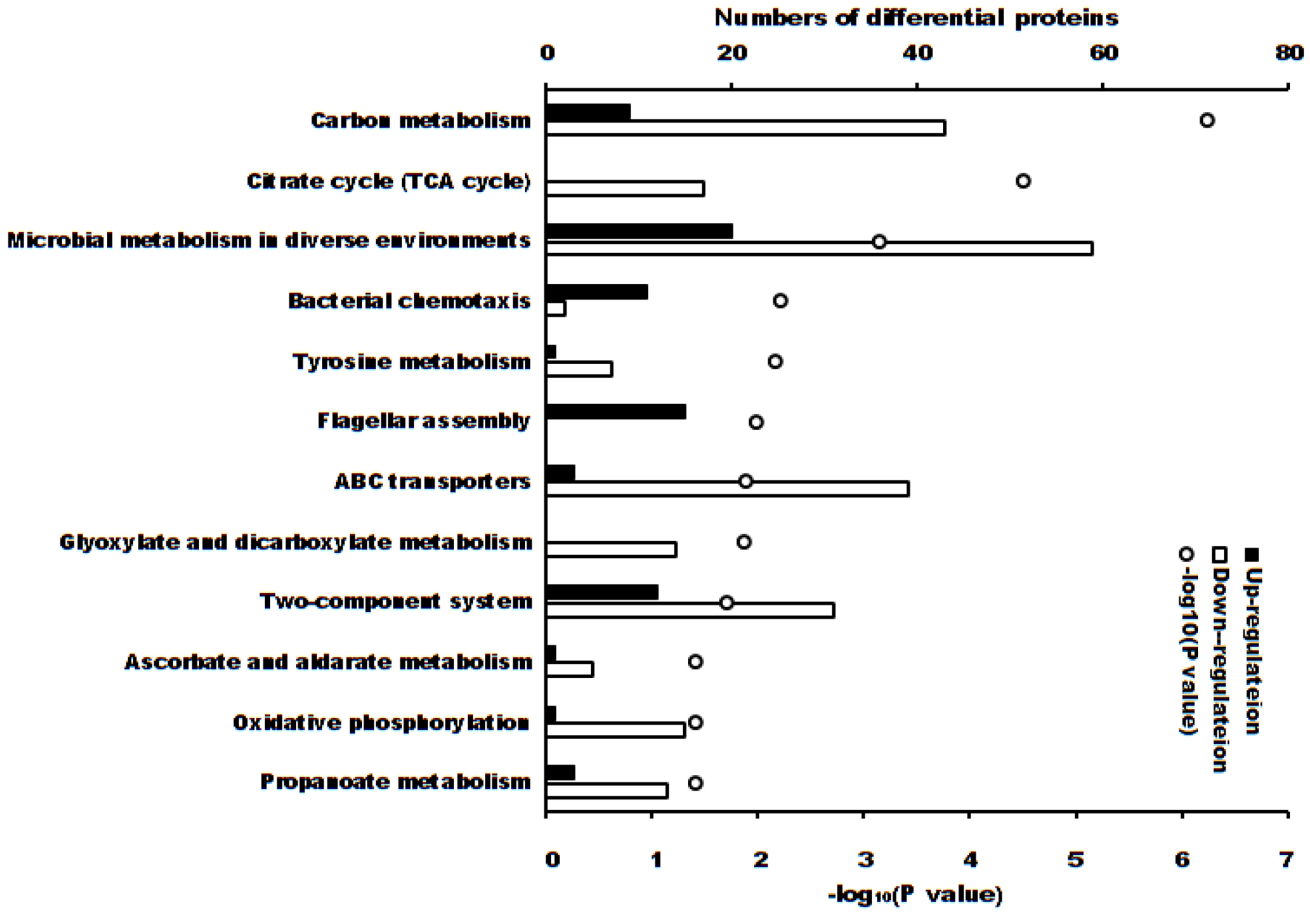
KEGG pathways (*p* < 0.05) of differential proteins between *S. Typhimurium* in ALH1 and control groups. The blank and solid bars mean down- or up-regulated differential proteins, repetitively. The open circle means –log_10_ (*p*-value).

These differentially expressed proteins were significantly enriched in 12 KEGG pathways (Figures 4, *p* < 0.05). There were 3 KEGG pathways if the adjusted *p-*value was considered: carbon metabolism (52 differential proteins, adjusted *p* = 5.47 × 10^−5^), citrate cycle (TCA cycle) (17 differential proteins, adjusted *p* = 1.47 × 10^−3^) and microbial metabolism in diverse environments (79 differential proteins, adjusted *p* = 0.0221).

**Figure 5:**
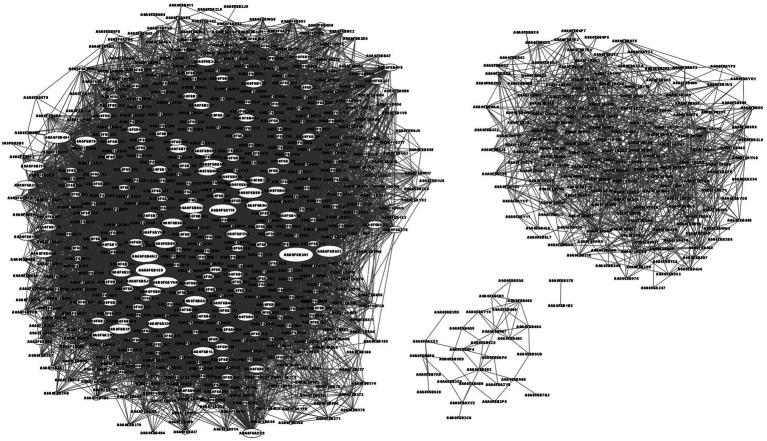
Network of protein–protein interactions. The characters in oval means protein ID. The line means the interaction of two proteins. The bigger size of the oval means more proteins interacted.

The protein–protein interaction network analysis is shown in Figure 5. The top 4 interacting proteins were aldehyde-alcohol dehydrogenase (A0A0F6B241, upregulated, *p* = 0.000158), putative pyruvate-flavodoxin oxidoreductase (A0A0F6B1S9, downregulated, *p* = 0.012980), N-acetyl glucosamine-specific PTS system components IIABC (A0A0F6AYI0, downregulated, *p* = 0.014904) and malic enzyme (A0A0F6B4M2, downregulated, *p* = 0.006444).

### 3.5. RT-PCR results

**Figure 6:**
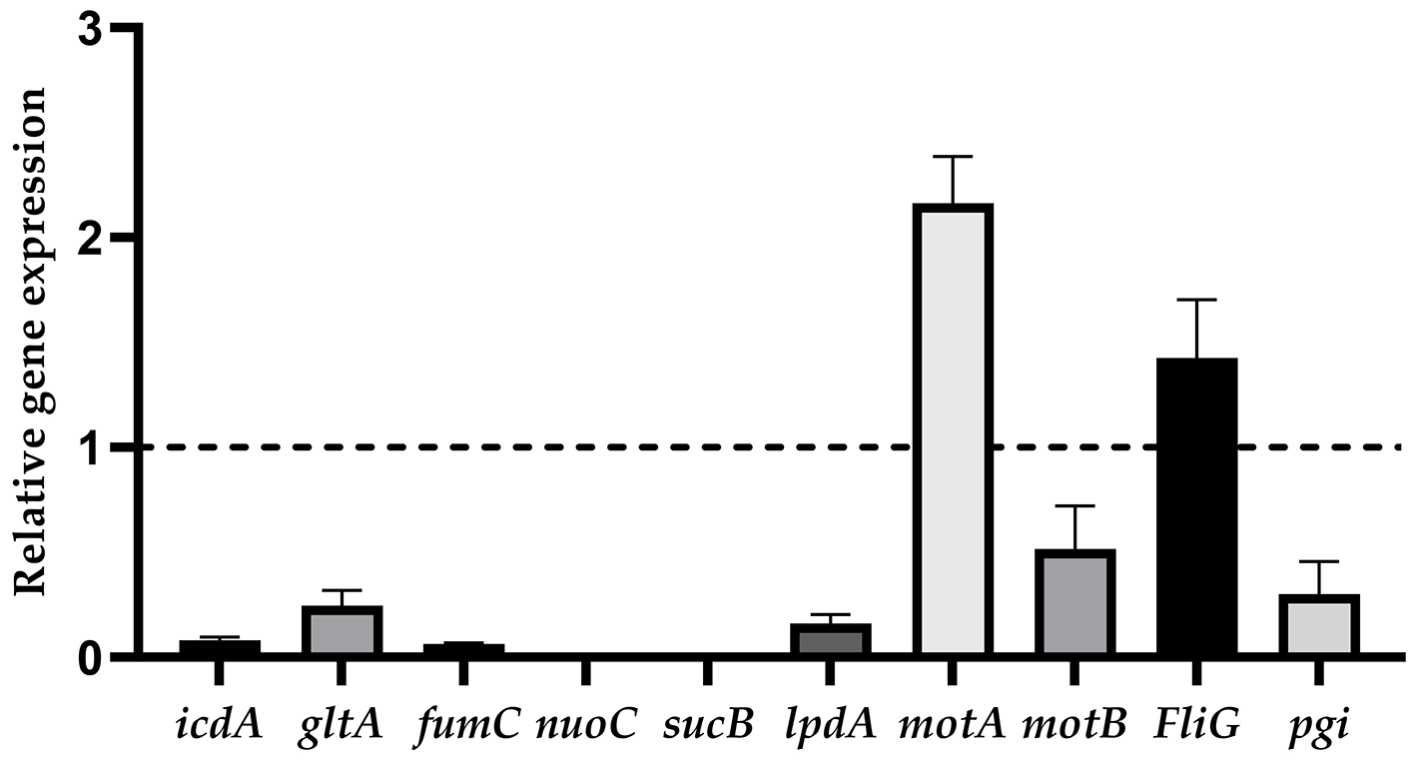
The relative expression of genes (ratio of the target gene to the internal reference gene).

The results showed that the relative expression levels of *motA* and *FliG* were upregulated, and *icdA*, *gltA*, *fumC*, *nuoC*, *sucB*, *lpdA*, *motB,* and *pgi* were downregulated (Figure 6).

## 4. Discussion

The physicochemical parameters of the ALH samples varied over a large range. These compounds were within the range in previous reports (17, 24, 25). The moisture content varied from 16.96 to 26.41%, which was influenced by the geographic locations, which have different relative humidity of air (1, 44) and maturity degrees (45). The relative humidity of air and maturity degrees of ALH were not controlled by us because the migratory habit of *Apis laboriosa*. Other parameters of samples with higher moisture, showed in dry weight, were in reasonable scopes (Table 3). Other parameters also had significant differences, which is in line with our previous report (24). They are affected by botanical sources, geographic locations and storage or transport conditions (24, 45–47). The different physicochemical parameters caused different antioxidant activities when they were determined using different assays (Table 5). However, except for O_2_·-, they have a high correlation (Table 6). Among these parameters, TPC and TFC were highly correlated with antioxidant activity (Table 6) determined using DPPḤ, ABTS· + and FRAP assays, agreed with other references (8, 12–15). The result of the superoxide anion scavenging activity assay was not in line with those of other antioxidant assays, which was also found in a previous report (48). These differences may be caused by different chemical components in honey and their detection principle (11). It was reported that TFC accounted for approximately 25–70% of TPC depending on the honey botanical source (49), which in ALH samples were 23–44%, except for that in ALH5. The high moisture content in ALH5 indicates a low ripening degree (45), which resulted in a low TFC.

The TPC and TFC in honey were also responsible for its antibacterial activity. Polyphenols with pyrogallol groups (50), methyl gallate and gallic acid (51, 52) showed strong antibacterial activity against various kinds of bacteria. Polyphenols with pyrogallol groups had strong antibacterial activity against 26 species of bacteria, but those with catechol and resorcinol rings showed lower activity (50). The MBC of methyl gallate and gallic acid against different bacteria varied from 0.5 to less than 8 mg/ml (51, 52). The MIC against *S.* Typhimurium varied from 20 to 30%, and the MBC varied from 25 to 40% ALH, with TPCs of 49–123 mg GAE/100 g (Table 3). The calculated TPCs in MIC or MBC were close to these reports (51, 52), even though the data were determined using differential species of bacteria. The antibacterial activities of honey depend on the type of flowers foraged by the bees and its geographical origin rather than its monofloral or polyfloral nature (53). However, there was no correlation between TPC and the well diffusion assay for *S. aureus* (54). The antibacterial activity of honey is attributed to many factors, including sugar, polyphenol compounds, hydrogen peroxide, 1, 2-dicarbonyl compounds, and bee defensin-1 contents (18). The phenolic compositions varied depending on the botanical sources of the ALH samples (Table 4), causing different antibacterial activities against *S.* Typhimurium.

The antibacterial activity can be determined using MIC, MBC or IC_50_, etc. For further proteomic experiments, the IC_50_ of ALH1 against *S.* Typhimurium was determined to be 2.97% (w/v) after 24 h of treatment (Figure 1). Then, this concentration was used for proteomic and RT–PCR experiments to determine the possible mechanism of the antibacterial activity. The differential proteins in *S.* Typhimurium between the ALH1-treated and control groups played an important role in its antibacterial activity. There were 160 differential proteins with *p* = 0 (Supplementary Table S2), of which there were 14 upregulated proteins. All the differential proteins were significantly enriched in 3 KEGG pathways (adjusted *p* < 0.05): carbon metabolism, TCA cycle and microbial metabolism in diverse environments.

First, carbon metabolism (adjusted *p* = 5.47 × 10^−5^) included 52 differential proteins (9 up- and 43 downregulations), whose proportion in total proteins of the pathway was 50.49% (Figure 4). There are six key oxidoreductases of central carbon metabolism (CCM): glucose-6-phosphate dehydrogenase, pyruvate dehydrogenase, 2-ketoglutarate dehydrogenase, malate dehydrogenase, malic enzyme, and isocitrate dehydrogenase (55). In this experiment, malate dehydrogenase (*p* = 0.00763), malic enzyme (*p* = 0.00644) and isocitrate dehydrogenase (*p* = 0.0113) were downregulated, which indicated that ALH1 inhibited central carbon metabolism *via* a decrease in oxidoreductase activity. Malic enzyme and isocitrate dehydrogenase are necessary for the normal growth and/or persistence of bacteria (56). ALH1 repressed central carbon metabolism and inhibited proliferation and survival *via* its antioxidant substances. The irreversible reaction glycolysis (one of CCM pathways) is glucokinase, which was downregulated (*p* = 0.00384). One of the key enzymes in the pentose phosphate pathway (PPP) of CCM, the downregulated transketolase (*p* = 0.045), decreased the process of glucose turnover that produces NADPH as reducing equivalents and pentoses as essential parts of nucleotides. Other key enzymes, such as glucose 6-phosphate dehydrogenase, 6-phosphogluconate dehydrogenase and NOX_2_ (57), were not differentially expressed proteins. Triosephosphate isomerase and phosphogluconate dehydratase were the only two proteins that were upregulated in the Entner-Doudoroff pathway to speed up the metabolism of glucose (58). There were no differential proteins in the semi or non-phosphorylative Entner-Doudoroff pathway. Another important part of CCM was the TCA cycle, which was also an enrichment pathway parallel to carbon metabolism.

The TCA cycle (adjusted *p* = 1.47 × 10^−3^) included 17 differentially expressed proteins (total downregulation, 13 differentially expressed proteins with *p* = 0), and the proportion of total proteins in the pathway was 68% (Figure 4). The TCA cycle, which is also one part of CCM, is a core process in aerobic respiration for energy production and the production of carbon-based precursor molecules for many biosynthetic pathways in aerobic organisms. It is also of vital importance for the survival of lifeforms (59). There are three key regulators of the catalytic reaction in the TAC cycle, citrate synthase, isocitrate dehydrogenase and α-ketoglutarate dehydrogenase (α-KGDH), because their reactions are irreversible (60). Citrate synthase, which catalyzes the synthesis of citrate from acetyl-CoA and oxaloacetate, is the first step of the TCA cycle (54). In this experiment, citrate synthase was downregulated in ALH1-treated *S.* Typhimurium (*p* = 0.0398), which decreased the speed of citrate synthesis. Another key regulator in the TAC cycle, isocitrate dehydrogenase, was also downregulated (*p* = 0.0113). Therefore, the amount of alpha-ketoglutarate converted to succinyl-CoA was decreased. These two downregulated key regulators decreased respiration for energy production and production of carbon-based precursor molecules in the TCA cycle, which indicates that ALH1 can effectively inhibit the oxidative respiratory metabolism of *S.* Typhimurium through the TCA pathway inhibiting bacterial survival. This result agreed with that of a previous report, such as that oregano essential oil against methicillin-resistant *S. aureus* decreased citrate synthase, isocitrate dehydrogenase and α-KGDH (61), Ag^+^ against *E. coli* decreased isocitrate dehydrogenase (62), kendomycin against methicillin-resistant *S. aureus* decreased α-ketoglutarate dehydrogenase (63), and clove essential oil against *S. aureus* decreased citrate synthase, isocitrate dehydrogenase and α-KGDH (64). Other carbohydrate metabolism pathways, including the glyoxylate cycle, ethylmalonyl pathway, methylaspartate cycle, photorespiration, malonate semialdehyde pathway, and propanoyl-CoA metabolism, were enriched in multiple downregulated and not upregulated proteins.

Additionally, carbon fixation pathways have multiple submodules. One of them was the phosphate acetyltransferase-acetate kinase pathway. Acetyl-CoA was catalyzed by the upregulated acetate kinase (*p* = 0.00119) and phosphate acetyltransferase (*p* = 7.23 × 10^−5^) to provide more ATP (65), and their reversibility enhances the use of acetate as the carbon source to be crucial for growth and substrate assimilation (66). These two enzymes were also involved in the methanogenesis pathway in methane metabolism. These results indicated that energy for bacteria treated with ALH1 was supplied *via* the phosphate acetyltransferase-acetate kinase pathway when the TCA cycle was inhibited. The upregulated methylenetetrahydrofolate reductase in the reductive acetyl-CoA pathway (wood-Ljungdahl pathway), which is the rate-limiting enzyme in the methyl cycle, increasingly committed tetrahydrofolate-bound one-carbon units to use in the methylation of homocysteine to form methionine (67). In methane metabolism, another upregulated protein was ATP-dependent 6-phosphofructokinase in formaldehyde assimilation, the ribulose monophosphate pathway, which catalyzes the phosphorylation of d-fructose 6-phosphate to fructose 1, 6-bisphosphate by ATP as the first committing step, the key regulatory step of glycolysis (68). Other differentially expressed proteins in carbon fixation pathways and methane metabolism were downregulated. There were 4 differentially expressed proteins in amino acid metabolism, which were downregulated to inhibit transporters for uptake, biosynthesis and degradation and extraction of amino acids.

The third significantly different pathway (adjusted *p* = 0.0221) was microbial metabolism in diverse environments, which had 20 up- and 59 downregulated proteins and 38.73% of total proteins in the pathway. Metabolism diversity reflects different metabolic strategies of bacteria to adapt to different environments and obtain the energy and carbon needed for the production of cellular constituents necessary for growth, survival, and reproduction. All of the differential proteins in this pathway were also enriched in other pathways because many of the compounds played an important role in both catabolic and anabolic reactions in cells (69). Therefore, differentially expressed proteins interact with each other.

The top 4 interacting proteins were aldehyde-alcohol, putative pyruvate-flavodoxin oxidoreductase, *N*-acetyl glucosamine specific PTS system components IIABC and malic enzyme (Figure 5). Aldehyde-alcohol dehydrogenase is a NAD(H)-dependent bifunctional and highly conserved enzyme in bacteria. This is essential for the fermentation of glucose to sustain the glycolytic pathway of acetyl-CoA to ethanol (70–72), utilization of ethanol as a substrate to generate NADH and other carbon intermediates (71, 72), and a functional enzyme such as pyruvate formate-lyase to catalyze the conversion of pyruvate and coenzyme A to formate and acetyl-CoA (70). Due to its multifunctional activity, aldehyde-alcohol dehydrogenase has the most interacting proteins. Putative pyruvate-flavodoxin oxidoreductase was enriched in glycolysis/gluconeogenesis, the citrate cycle (TCA cycle), pyruvate metabolism, butanoate metabolism, metabolic pathways, the biosynthesis of secondary metabolites, microbial metabolism in diverse environments and carbon metabolism pathways to produce acetyl-CoA from pyruvate (73, 74). *N*-acetyl glucosamine-specific PTS system component IIABC was enriched in amino sugar and nucleotide sugar metabolism and the phosphotransferase system (PTS) (75). Malic enzyme, a major contributor to cellular reducing power and carbon flux, was engaged in NADP - malic enzyme type C4-dicarboxylic acid cycle, pyruvate metabolism, metabolic pathways and microbial metabolism in diverse environments to fit the new component in culture medium (76). The number of interacting proteins were determined by the complexity of the metabolic system and the pathway it engaged.

The different relative expressions of proteins were not the same as the relative expression trend of genes selected to undergo RT-PCR (Figure 6). The relative expression of genes is influenced by posttranscriptional regulation and stability of mRNA (77).

There are some defects in this research on the possible mechanism of ALH1 antibacterial activity against *S.* Typhimurium but other ALH samples were not employed for this purpose because of the high cost of the proteomic experiment. It is difficult to refer to the key or an absolute advantage pathway to explain the possible mechanism because there were too many differential proteins obtained and too much diversity in the metabolism of bacteria. In particular, carbon metabolism has 47 submodules, and proteins participate in more than 1 or 2 pathways. Therefore, the C^13^-carboxyl-labeled method can be used to confirm the mechanism precisely. Metabolomics, single components separated from phenols or flavonoids as treatments, single-cell transcriptomics, deletion genes of key proteins, or other methods can be employed to more accurately explore the regulation of ALH against *S.* Typhimurium.

## 5. Conclusion

The different antioxidant activities of ALH samples harvested at different times and from different regions were affected by their physicochemical parameters, especially TPC and TFC, which have a high correlation between TPC or TFC and antioxidant activity except for the O_2_·- assay. The MIC and MBC of ALH against *S.* Typhimurium were 20–30% and 25–40%, respectively. Proteomic analysis revealed the possible antibacterial mechanism of ALH1 at IC_50_ (2.97%, w/v), whose antioxidant activity can reduce the bacterial reduction reaction and energy supply, inhibit the TCA cycle and amino acid metabolism and enhance glycolysis. Overall, the results provide a theoretical basis for the treatment of *S.* Typhimurium using ALH.

## Data availability statement

The original contributions presented in the study are publicly available. This data can be found here: https://www.iprox.cn//page/project.html?id=IPX0006322000.

## Author contributions

HK and WY: conceptualization. WT, YT, and QZ: methodology. WT and YT: formal analysis. HK and SM: investigation and resources. WT and WW: writing – original draft preparation. WY: writing – review and editing, project administration, and funding acquisition. WY and XM: supervision. All authors have read and agreed to the published version of the manuscript.

## Funding

This research was funded by Fujian Provincial Natural Science Funding for WY, grant number 2020 J01540.

## Conflict of interest

The authors declare that the research was conducted in the absence of any commercial or financial relationships that could be construed as a potential conflict of interest.

## Publisher’s note

All claims expressed in this article are solely those of the authors and do not necessarily represent those of their affiliated organizations, or those of the publisher, the editors and the reviewers. Any product that may be evaluated in this article, or claim that may be made by its manufacturer, is not guaranteed or endorsed by the publisher.

## Footnotes

^1^https://www.mzcloud.org/
