# Supplementary material for: Antioxidant and antibacterial activity of Apis laboriosa honey against Salmonella enterica serovar Typhimurium
Source: Front Nutr. 2023 May 11;10:1181492. doi: 10.3389/fnut.2023.1181492 (PMC10211265; doi:10.3389/fnut.2023.1181492)
Supplement: Supplementary file 1 [file Data_Sheet_1.docx]

Supplementary Material

Antioxidant and Antibacterial Activity of *Apis Laboriosa* Honey against *Salmonella enterica* serovar Typhimurium

Weihua Tan ^2, 4Ϯ^, Yuanyuan Tian ^1, 4Ϯ^, Qingya Zhang ^2, 4^, Siwei Miao ^3^, Wenrong Wu^2, 4^, Xiaoqing Miao ^3, 4^, Haiou Kuang ^3, 5^ and Wenchao Yang ^1, 2, 3, 4^*

*** Correspondence:** Wenchao Yang 000q061005@fafu.edu.cn

# Supplement Table S1

**Table S1** Primer sequence for RT-PCR

| Primer | Sequence（5'→3'） |
| --- | --- |
| 5s -F | TTGTCTGGCGGCAGTAGC |
| 5s-R | TTTGATGCCTGGCAGTTC |
| icdA-F | CCCGCTGAAACCCTTGATT |
| icdA-R | ACTTGGCGTGCCCTGATAG |
| gltA-F | CCACGAAGTGCTGAAAGAG |
| gltA-R | AATGATGCCGGAGTAGAAG |
| fumc-F | TGCTATGTTGCCAGGTGAT |
| fumc-R | TTCCTGACGCACAGTGTTTA |
| pgi-F | TCACCACCCAGGAAACCAT |
| pgi-R | GAGTAACGACCACCGACCC |
| nuoC-F | ACCAGCGACGGCAGCACTA |
| nuoC-R | GCGGTCCACATCTGACATAAC |
| SucB-F | AGGCGAGTCTAAAGCACCC |
| SucB-R | TCATTGAAGGTCGTCAGCAT |
| lpdA-F | GCCGTAAAGTGAAAGTGGTC |
| lpdA-R | AGAGCGTCGGTAGAATCCC |
| motA-F | AAATCTTCGCCAGTTATCC |
| motA-R | CTTCATCCATCAACGCTTC |
| motB-F | CCATACCGATGACTTTCCC |
| motB-R | GCCAACCACCCGTAATACTT |
| FliG-F | ATCAACGCCAACGAATACC |
| FliG-R | ATCAACGCCAACGAATACC |

# Supplementary Figures

**Figure S1 Chromatographic profiles determined by UPLC-Q-TOF-M**

(A) Positive mode

Blank

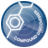

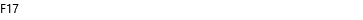

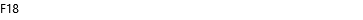

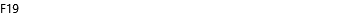

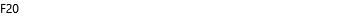

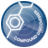

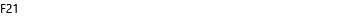

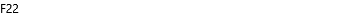
QC1

QC2

QC3

ALH1

ALH2

ALH3

ALH4

ALH5

(B) Negative mode

Blank

QC1

QC2

QC3

ALH1

ALH2

ALH3

ALH4

ALH5

# Supplement Table S2

**Table S2** Differential proteins with the highest significant (p = 0)

| **Protein ID** | **Description** | **up/down-regulation** | **Enriched GO_term** | **Enriched KEGG_pathway** |
| --- | --- | --- | --- | --- |
| A0A0F6B259 | Nitrate/nitrite transporter | up | Integral component of membrane, Transmembrane transport | Nitrogen metabolism |
| A0A0F6B2W4 | Flagellar biosynthetic protein FliP | up | Protein secretion, Membrane | Flagellar assembly |
| A0A0F6B013 | Flagellar basal body protein | up | Bacterial-type flagellum-dependent cell motility | Flagellar assembly |
| A0A0F6B1D8 | Putative outer membrane protein | up | Transport, Porin activity, Membrane | --***** |
| A0A0F6B573 | tRNA1(Val) (adenine(37)-N6)-methyltransferase | up | Methyltransferase activity | -- |
| A0A0F6AXX6 | Putative transport protein | up | Integral component of membrane, Transmembrane transport | -- |
| A0A0F6B804 | Low affinity gluconate transporter | up | Gluconate transmembrane transporter activity, Membrane, Gluconate transmembrane transport | -- |
| A0A0F6B6V6 | Sensor protein QseC | down | Phosphorelay sensor kinase activity, Signal transduction | Two-component system, Quorum sensing |
| A0A0F6B891 | C4-dicarboxylate transport protein | down | Symporter activity, Integral component of membrane | Two-component system |
| A0A0F6AY72 | Outer membrane receptor FepA | down | Receptor activity, Transport, Membrane | Two-component system |
| A0A0F6B5I5 | Regulatory protein | down | Phosphorelay signal transduction system, DNA binding | Two-component system |
| A0A0F6B0Y1 | Response regulator | down | Phosphorelay signal transduction system | Two-component system |
| A0A0F6B0Y0 | Histidine kinase | down | Phosphorelay sensor kinase activity, Signal transduction | Two-component system |
| A0A0F6B4E5 | Histidine kinase | down | Phosphorelay sensor kinase activity, Signal transducer activity, Signal transduction, Integral component of membrane | Two-component system |
| A0A0F6B5I6 | Tricarboxylic transport | down | Outer membrane-bounded periplasmic space | Two-component system |
| A0A0F6AWJ0 | Probable 2-(5-triphosphoribosyl)-3-dephosphocoenzyme-A synthase | down | ATP binding, Phosphorylation, Triphosphoribosyl-dephospho-CoA synthase activity | Two-component system |
| A0A0F6BAG6 | Putative anaerobic dimethylsulfoxide reductase subunit B | down | -- | Sulfur metabolism |
| A0A0F6B2U7 | Cytoplasmic alpha-amylase | down | Catalytic activity, Carbohydrate metabolic process, Cation binding | Starch and sucrose metabolism |
| A0A0F6B4L5 | Phosphate acetyltransferase | down | Metabolic process | Pyruvate metabolism |
| A0A0F6B397 | Propanol dehydrogenase | down | Oxidoreductase activity, Metal ion binding, Oxidation-reduction process | Propanoate metabolism |
| A0A0F6AXI1 | 2-methylcitrate dehydratase | down | Lyase activity | Propanoate metabolism |
| A0A0F6B386 | Propanediol dehydratase medium subunit | down | -- | Propanoate metabolism |
| A0A0F6AXI0 | Citrate synthase | down | -- | Propanoate metabolism |
| A0A0F6AXH9 | 2-methylisocitrate lyase | down | -- | Propanoate metabolism |
| A0A0F6B374 | Cobalt-precorrin-6Y C(15)-methyltransferase | down | Methyltransferase activity | Porphyrin and chlorophyll metabolism |
| A0A0F6B377 | Precorrin-8X methylmutase | down | Cobalamin biosynthetic process, Precorrin-8X methylmutase activity | Porphyrin and chlorophyll metabolism |
| A0A0F6B378 | Cobalamin biosynthesis protein CbiB | down | Cobalamin biosynthetic process, Integral component of membrane, Threonine-phosphate decarboxylase activity | Porphyrin and chlorophyll metabolism |
| A0A0F6B8U0 | Putative PTS system galactitol-specific enzyme IIC component | down | Phosphoenolpyruvate-dependent sugar phosphotransferase system, Integral component of membrane | Phosphotransferase system (PTS) |
| A0A0F6BB78 | Putative PTS permease | down | Cytoplasm, Protein-N(PI)-phosphohistidine-sugar phosphotransferase activity, Phosphoenolpyruvate-dependent sugar phosphotransferase system | Phosphotransferase system (PTS) |
| A0A0F6AWN7 | L-arabinose isomerase | down | Metabolic process, L-arabinose isomerase activity | Pentose and glucuronate interconversions |
| A0A0F6BB45 | Putative aspartate racemase | down | Nitrogen compound metabolic process | Nonribosomal peptide structures, Alanine, aspartate and glutamate metabolism |
| A0A0F6B5J2 | Succinate-semialdehyde dehydrogenase I | down | Metabolic process, Oxidoreductase activity, Oxidation-reduction process | Microbial metabolism in diverse environments, Tyrosine metabolism, Lysine degradation, Alanine, aspartate and glutamate metabolism |
| A0A0F6B3N7 | Putative flutathione S-transferase | down | Catalytic activity, Metabolic process | Microbial metabolism in diverse environments, Tyrosine metabolism |
| A0A0F6B3N8 | Putative 1,2-dioxygenase | down | -- | Microbial metabolism in diverse environments, Tyrosine metabolism |
| A0A0F6B1J1 | Nitrate reductase (quinone) | down | Oxidoreductase activity, Molybdenum ion binding, Oxidation-reduction process | Microbial metabolism in diverse environments, Two-component system, Nitrogen metabolism |
| A0A0F6B618 | Sulfite reductase [NADPH] hemoprotein beta-component | down | Oxidoreductase activity, Heme binding, Iron-sulfur cluster binding, Oxidation-reduction process | Microbial metabolism in diverse environments, Sulfur metabolism |
| A0A0F6B605 | Sulfate adenylyltransferase subunit 2 | down | Catalytic activity, Metabolic process | Microbial metabolism in diverse environments, Sulfur metabolism |
| A0A0F6BAD0 | Cytochrome c-552 | down | Nitrogen compound metabolic process, Periplasmic space, Oxidation-reduction process | Microbial metabolism in diverse environments, Salmonella infection, Nitrogen metabolism |
| A0A0F6B1E6 | Putative hydrogenase-1 large subunit | down | Nickel cation binding | Microbial metabolism in diverse environments, Nitrotoluene degradation |
| A0A0F6B1E7 | NiFe hydrogenase | down | Iron-sulfur cluster binding, Oxidation-reduction process | Microbial metabolism in diverse environments, Nitrotoluene degradation |
| A0A0F6B487 | Putative phosphotransferase system enzyme II A component | down | -- | Microbial metabolism in diverse environments, Ascorbate and aldarate metabolism, Phosphotransferase system (PTS) |
| A0A0F6BAQ1 | 3-keto-L-gulonate-6-phosphate decarboxylase UlaD | down | Orotidine-5'-phosphate decarboxylase activity, 'De novo' pyrimidine nucleobase biosynthetic process | Microbial metabolism in diverse environments, Ascorbate and aldarate metabolism, Pentose and glucuronate interconversions |
| A0A0F6B8T3 | Putative glycosyl hydrolase | down | Carbohydrate metabolic process | Glycolysis / Gluconeogenesis, Starch and sucrose metabolism |
| A0A0F6B5P3 | Glucitol/sorbitol-specific enzyme IIB component | down | Protein-N(PI)-phosphohistidine-sugar phosphotransferase activity, Phosphoenolpyruvate-dependent sugar phosphotransferase system , Integral component of membrane | Fructose and mannose metabolism, Phosphotransferase system (PTS) |
| A0A0F6B5P5 | Sorbitol-6-phosphate dehydrogenase | down | -- | Fructose and mannose metabolism |
| A0A0F6B9R0 | Putative epimerase LsrE | down | Carbohydrate metabolic process | Carbon metabolism,Microbial metabolism in diverse environments, Pentose and glucuronate interconversions, Pentose phosphate pathway |
| A0A0F6B6A8 | Acetyl-CoA acetyltransferase | down | Metabolic process | Carbon metabolism, Microbial metabolism in diverse environments, Glyoxylate and dicarboxylate metabolism, Two-component system, Propanoate metabolism, Butanoate metabolism, Tryptophan metabolism, Valine, leucine and isoleucine degradation, Lysine degradation, Fatty acid degradation, Benzoate degradation |
| A0A0F6B9K5 | Formate dehydrogenase-O subunit gamma | down | Electron carrier activity, Integral component of membrane | Carbon metabolism, Microbial metabolism in diverse environments, Glyoxylate and dicarboxylate metabolism, Methane metabolism |
| A0A0F6BA19 | Isocitrase | down | Isocitrate lyase activity, Carboxylic acid metabolic process | Carbon metabolism, Microbial metabolism in diverse environments, Glyoxylate and dicarboxylate metabolism |
| A0A0F6B731 | L-threonine dehydratase catabolic TdcB | down | -- | Carbon metabolism, Glycine, serine and threonine metabolism |
| A0A0F6AYN2 | Succinate dehydrogenase cytochrome b556 subunit | down | -- | Carbon metabolism, Citrate cycle (TCA cycle), Microbial metabolism in diverse environments, Oxidative phosphorylation, Butanoate metabolism |
| A0A0F6AYD8 | Peptidoglycan D,D-transpeptidase MrdA | down | Penicillin binding | beta-Lactam resistance |
| A0A0F6B0N7 | N-succinylarginine dihydrolase | down | Arginine metabolic process, N-succinylarginine dihydrolase activity | Arginine and proline metabolism |
| A0A0F6B0N5 | Arginine N-succinyltransferase | down | Arginine catabolic process, Arginine N-succinyltransferase activity | Arginine and proline metabolism |
| A0A0F6B0X4 | Putative proline iminopeptidase | down | -- | Arginine and proline metabolism |
| A0A0F6AYF9 | Glutamate/aspartate transporter | down | ATP binding, ATPase activity | ABC transporters, Two-component system |
| A0A0F6B1V8 | Periplasmic murein tripeptide transport protein | down | -- | ABC transporters, beta-Lactam resistance, Quorum sensing |
| A0A0F6B3P8 | Beta-methylgalactoside transporter inner membrane component | down | Transporter activity, Transport, Membrane | ABC transporters |
| A0A0F6B195 | Putative ABC transporter periplasmic component | down | Transporter activity, Transport | ABC transporters |
| A0A0F6BA73 | Maltose/maltodextrin transporter ATP-binding protein | down | Transporter activity, ATP binding, Transport, ATPase activity, ATP-binding cassette (ABC) transporter complex | ABC transporters |
| A0A0F6AZ16 | Putative ABC transporter periplasmic binding protein | down | Transport, Membrane | ABC transporters |
| A0A0F6B496 | Histidine/lysine/arginine/ornithine transport protein | down | Transport, Membrane | ABC transporters |
| A0A0F6B924 | Phosphate transport system permease protein PstA | down | Transport, Membrane | ABC transporters |
| A0A0F6B8B0 | Dipeptide transporter ATP-binding subunit | down | Nucleotide binding, ATP binding, Peptide transport, ATPase activity | ABC transporters |
| A0A0F6B5S8 | Putative periplasmic binding protein | down | Metal ion transport, Mtal ion binding | ABC transporters |
| A0A0F6B3M0 | Putative ABC-type proline/glycine betaine transport system ATPase component | down | ATP binding, ATPase activity | ABC transporters |
| A0A0F6B5S9 | Putative ATP-binding protein | down | ATP binding, ATPase activity | ABC transporters |
| A0A0F6B499 | Lysine/arginine/ornithine transport protein | down | -- | ABC transporters |
| A0A0F6AZ49 | Putrescine-binding periplasmic protein | down | -- | ABC transporters |
| A0A0F6B669 | Amino-acid acetyltransferase | down | N-acetyltransferase activity | 2-Oxocarboxylic acid metabolism |
| A0A0F6B9W0 | N-acetyl-gamma-glutamyl-phosphate reductase | down | N-acetyl-gamma-glutamyl-phosphate reductase activity, Cytoplasm, Cellular amino acid biosynthetic process, Protein dimerization activity, NAD binding, Oxidation-reduction process | 2-Oxocarboxylic acid metabolism |
| A0A0F6B9R8 | Glycerol diffusion | down | Transporter activity, Transport,membrane | -- |
| A0A0F6BAC6 | Cation/acetate symporter ActP | down | Transporter activity, Transport, Membrane, Transmembrane transport | -- |
| A0A0F6B359 | Uncharacterized protein | down | Transferase activity | -- |
| A0A0F6B052 | Putative periplasmic protein | down | Transferase activity | -- |
| A0A0F6B381 | Transcriptional regulator | down | Sequence-specific DNA binding | -- |
| A0A0F6B5V7 | Surface presentation of antigens protein SpaP | down | Protein secretion, Membrane | -- |
| A0A0F6B6X3 | Thiol:disulfide interchange protein DsbL | down | Protein disulfide oxidoreductase activity | -- |
| A0A0F6B3X9 | Cytochrome c-type biogenesis protein CcmE | down | Plasma membrane, Protein-heme linkage, Cytochrome complex assembly | -- |
| A0A0F6B8R3 | Putative transcriptional regulator | down | Phosphorelay signal transduction system, DNA binding | -- |
| A0A0F6B3L6 | Histidine kinase | down | Phosphorelay sensor kinase activity, Phosphorelay signal transduction system, Protein histidine kinase activity, Protein binding , integral component of membrane, Cell wall organization | -- |
| A0A0F6B9N8 | Periplasmic repressor CpxP | down | Periplasmic space | -- |
| A0A0F6B667 | Membrane-bound lytic murein transglycosylase A | down | Peptidoglycan turnover, Outer membrane | -- |
| A0A0F6B5W7 | Needle complex outer membrane lipoprotein | down | Pathogenesis | -- |
| A0A0F6B3X7 | Cytochrome c biogenesis protein CcmG | down | Oxidoreductase activity | -- |
| A0A0F6AYA6 | Putative dehydrogenase | down | Oxidation-reduction process | -- |
| A0A0F6B0R6 | Endonuclease | down | Nucleic acid binding, Hydrolase activity, Metal ion binding | -- |
| A0A0F6B1P3 | Aminoglycoside N(6)-acetyltransferase type 1 | down | N-acetyltransferase activity | -- |
| A0A0F6B4J9 | N-acetylmuramoyl-L-alanine amidase | down | N-acetylmuramoyl-L-alanine amidase activity, Peptidoglycan catabolic process | -- |
| A0A0F6AWG0 | Putative arylsulfatase | down | Metabolic process,Sulfuric ester hydrolase activity | -- |
| A0A0F6AXZ1 | Putative inner membrane protein | down | Membrane | -- |
| A0A0F6AY77 | Ferric enterobactin transport protein FepE | down | Lipopolysaccharide biosynthetic process, Membrane | -- |
| A0A0F6B1E1 | Putative hydrogenase | down | Iron ion binding | -- |
| A0A0F6B657 | DNA-binding transcriptional activator FucR | down | Intracellular | -- |
| A0A0F6B8I7 | L-lactate permease | down | Integral component of plasma membrane, Lactate transmembrane transporter activity,Lactate transport | -- |
| A0A0F6BAY7 | Putative arginine repressor | down | Integral component of membrane | -- |
| A0A0F6B703 | 2,4-dieonyl-CoA reductase | down | FMN binding, Oxidoreductase activity, Oxidation-reduction process | -- |
| A0A0F6B2Y3 | Putative cold-shock protein | down | DNA binding | -- |
| A0A0F6B699 | Putative transcriptional regulator | down | DNA binding | -- |
| A0A0F6B1I7 | Putative transcriptional regulator | down | DNA binding | -- |
| A0A0F6AZ83 | Cold shock-like protein CspD | down | DNA binding | -- |
| A0A0F6B9L7 | L-rhamnose mutarotase | down | Cytoplasm | -- |
| A0A0F6AY89 | Carbon starvation protein | down | Cellular response to starvation, Membrane | -- |
| A0A0F6AWR3 | Cell division protein FtsL | down | Cell cycle, Integral component of membrane,cell division | -- |
| A0A0F6AY31 | Fimbrial protein | down | Cell adhesion, Pilus | -- |
| A0A0F6B3K8 | Putative fimbrial-like protein | down | Cell adhesion, Pilus | -- |
| A0A0F6AWG6 | Putative glycosyl hydrolase | down | Carbohydrate metabolic process | -- |
| A0A0F6B869 | Anaerobic C4-dicarboxylate transporter | down | C4-dicarboxylate transmembrane transporter activity, C4-dicarboxylate transport, Integral component of membrane | -- |
| A0A0F6BB76 | Putative transcriptional regulator | down | ATP binding, Transcription factor binding, Phosphoenolpyruvate-dependent sugar phosphotransferase system, Integral component of membrane | -- |
| A0A0F6B4L8 | Putative ethanolamine utilization protein | down | ATP binding, Cellular biogenic amine metabolic process | -- |
| A0A0F6B1L3 | Putative virulence protein | up | -- | -- |
| A0A0F6B290 | Hydrogenase-1 operon protein HyaE | up | -- | -- |
| A0A0F6B886 | Anti-FlhC(2)FlhD(4) factor YdiV | up | -- | -- |
| A0A0F6AXZ3 | Putative outer membrane protein | up | -- | -- |
| A0A0F6B4Z6 | Putative ferredoxin | up | -- | -- |
| A0A0F6BBA2 | Nucleotidase | up | -- | -- |
| A0A0F6B7F4 | Putative periplasmic protein | up | -- | -- |
| A0A0F6B395 | Corrinoid adenosyltransferase | down | -- | -- |
| A0A0F6B1N6 | Putative cellulase protein | down | -- | -- |
| A0A0F6B0X5 | Putative cytoplasmic protein | down | -- | -- |
| A0A0F6B1Q0 | Putative transcriptional regulator | down | -- | -- |
| A0A0F6B2A3 | Alanine racemase | down | -- | -- |
| A0A0F6B3V9 | O-antigen acetylase | down | -- | -- |
| A0A0F6AWV8 | Putative inner membrane protein | down | -- | -- |
| A0A0F6B692 | Putative peptide transport protein | down | -- | -- |
| A0A0F6B1C0 | Competence damage-inducible protein A | down | -- | -- |
| A0A0F6BAX0 | 4-hydroxy-2-oxoglutarate aldolase | down | -- | -- |
| A0A0F6AY56 | Outer membrane esterase | down | -- | -- |
| A0A0F6B1H3 | Putative lipoprotein | down | -- | -- |
| A0A0F6BAE0 | TPR repeat-containing protein | down | -- | -- |
| A0A0F6AXS3 | Transcriptional regulator BolA | down | -- | -- |
| A0A0F6B870 | Putative sugar kinase | down | -- | -- |
| A0A0F6B3X6 | Cytochrome c-type biogenesis protein | down | -- | -- |
| A0A0F6BB36 | DUF302 domain-containing protein | down | -- | -- |
| A0A0F6B6P2 | Putative cytoplasmic protein | down | -- | -- |
| A0A0F6AYW5 | Cardiolipin synthase B | down | -- | -- |
| A0A0F6B0W8 | Cysteine desulfuration protein SufE | down | -- | -- |
| A0A0F6B5Y4 | AP_endonuc_2 domain-containing protein | down | -- | -- |
| A0A0F6B1G1 | Putative cytoplasmic protein | down | -- | -- |
| A0A0F6B6X2 | Arylsulfate sulfotransferase AssT | down | -- | -- |
| A0A0F6B4G2 | Putative negative regulator | down | -- | -- |
| A0A0F6B9Q0 | Putative mannose-6-phosphate isomerase | down | -- | -- |
| A0A0F6B813 | Putative cytoplasmic protein | down | -- | -- |
| A0A0F6B5Y6 | Putative tRNA synthase | down | -- | -- |
| A0A0F6AWL1 | Putative outer membrane lipoprotein | down | -- | -- |
| A0A0F6BBC3 | Uncharacterized protein | down | -- | -- |
| A0A0F6AYW8 | Transport permease protein | down | -- | -- |
| A0A0F6B901 | Putative mandelate racemase | down | -- | -- |
| A0A0F6B732 | DNA-binding transcriptional activator TdcA | down | -- | -- |
| A0A0F6B4R2 | Putative inner membrane protein | down | -- | -- |
| A0A0F6BB42 | Putative hyperinvasive locus E | down | -- | -- |
| A0A0F6B6X7 | Surface composition regulator | down | -- | -- |
| A0A0F6B399 | Polyhedral body protein | down | -- | -- |
| A0A0F6AXF5 | Putative outer membrane lipoprotein | down | -- | -- |
| A0A0F6AX34 | Ribonuclease HII | down | -- | -- |
| A0A0F6B5B5 | Putative cytoplasmic protein | down | -- | -- |
| A0A0F6B7B2 | Uncharacterized protein | down | -- | -- |
| A0A0F6B1E2 | Putative hydrogenase | down | -- | -- |
| A0A0F6AYW6 | Putative cytoplasmic protein | down | -- | -- |
| A0A0F6B0Q9 | Putative regulatory protein | down | -- | -- |
